# An IL-17A-centric response to Epstein-Barr virus DNA mediated by dendritic Cell-T cell interactions

**DOI:** 10.3389/fmolb.2024.1243366

**Published:** 2024-04-04

**Authors:** Marwa Shehab, Hadi Hussein, Sukayna Fadlallah, Elias A. Rahal

**Affiliations:** ^1^ Department of Experimental Pathology, Immunology and Microbiology, American University of Beirut, Beirut, Lebanon; ^2^ Center for Infectious Diseases Research, American University of Beirut, Beirut, Lebanon

**Keywords:** Epstein—Barr virus, IL-17A, Th17, endosomal toll like receptors, inflammation, autoimmunity, TLR3, TLR9

## Abstract

**Introduction:** The Epstein-Barr virus has been associated with a considerable number of autoimmune diseases. We have previously demonstrated that EBV DNA enhances the production of IL-17A, a pro-inflammatory cytokine, via endosomal Toll-like receptor signalling.

**Methods:** We used RNA-seq to analyze the transcriptional profile of mouse immune cells treated with EBV DNA.

**Results:** We observed that EBV DNA upregulates an IL-17A-centric network of mediators. Ensemble Gene Set Enrichment Analysis (EGSEA) showed enriched expression of sets involved in inflammatory responses including IFNγ and TNF-α-associated pathways as well as proinflammatory diseases. On the other hand, while macrophages and B cells were somewhat able to induce an IL-17A response from T cells to EBV DNA, they were less potent than dendritic cells. EBV virions were also capable of eliciting the production of inflammatory mediators from dendritic cell-T cell cultures largely mirroring responses to the viral DNA.

**Conclusions:** Given the wide prevalence of EBV in the population, our analyses reveal a network of mediators and cell types that may serve as therapeutic targets in a large proportion of people affected by autoimmune diseases.

## 1 Introduction

The Epstein-Barr virus (EBV) is known to be a potent modulator of immune processes. This virus establishes a lifelong latent infection with frequent reactivations in the host (Lunemann et al., 2007). Due to its ubiquitous nature, frequent reactivation potential and immune response modulation, EBV is thought to be involved in several autoimmune diseases. The general consensus is that EBV acts as an inflammatory trigger providing innate and adaptive activation signals (Iwakiri, 2014). EBV has been associated with autoimmune diseases including systemic lupus erythematosus (SLE) (James et al., 1997), rheumatoid arthritis (RA) (Lotz and Roudier, 1989), Sjorgen’s syndrome (SS) (Fox et al., 1987), and multiple sclerosis (MS) (Ascherio and Munger, 2010).

Ninety-nine percent of young SLE patients are seropositive for EBV compared to 70% of age-matched controls (James et al., 1997). Moreover, increased levels of IgA antibodies against the EBV viral capsid antigen (VCA) were detected in sera of SLE patients (Chen et al., 2005). On the other hand, several sero-epidemiological investigations reported a higher incidence of MS in EBV infected patients (Lunemann et al., 2007) and some studies have described increased titers of antibodies against EBV antigens in RA patient sera (Shirodaria et al., 1987). Higher levels of Epstein-Barr nuclear antigen 1 (EBNA-1) antibodies have also been observed in SLE, MS and RA patients (Petersen et al., 1990; Lunemann et al., 2008; Draborg et al., 2012).

An increase in latently infected memory B cells is believed to underlie an elevated EBV genome load in SLE patients compared to healthy controls (Kang et al., 2004; Gross et al., 2005; Larsen et al., 2011). Furthermore, there a direct association between the number of EBV infected B cells and SLE disease activity (Gross et al., 2005). A high EBV load has also been observed in RA patients (Tosato et al., 1984; Blaschke et al., 2000; Balandraud et al., 2003; Lunemann et al., 2008). In MS patients, inconsistent data regarding the EBV viral load has been reported; Wandinger et al. (2000) detected higher levels of EBV DNA in patient sera during disease exacerbation periods and Wagner et al. (2004) described an association between EBV DNA in plasma and an increased risk of MS. Other studies did not detect any differences in EBV DNA levels between blood samples from MS patients and controls (Lunemann et al., 2006; Lindsey et al., 2009; Lucas et al., 2011).

Given that EBV DNA is rich in immunostimulatory CpG motifs (Kieff et al., 1982; Fiola et al., 2010), we previously assessed its contribution to autoimmune disease and proinflammatory pathways. We observed not only increased levels of EBV DNA in blood from RA patients, but also a correlation between EBV DNA levels and those of IL-17A, a proinflammatory mediator produced by T helper 17 (Th17) cells and highly associated with autoimmune processes (Salloum et al., 2018). Administration of EBV DNA to mice and treatment of mouse peripheral blood mononuclear cells with the viral DNA also enhanced the production of IL-17A via endosomal Toll-like receptor (TLR) signalling (Rahal et al., 2015; Shehab et al., 2019). In the study at hand, we sought to examine the network of mediators involved as well as determine the cell types that play a role in this response.

## 2 Methods

### 2.1 RNA-seq and transcriptional profile analyses

To assess the effect of EBV DNA on the transcriptional profile of mouse PBMCs, RNA-seq was used. Study protocols were approved by the Animal Care and Use Committee (IACUC) at the American University of Beirut (AUB). BALB/c mouse PBMCs were isolated from 4 to 6 week old female mouse blood using Histopaque^®^ (Sigma-Aldrich, Saint Louis, Missouri) and then were cultured in a 96-well plate whereby each well contained 25 × 10^4^ PBMCs in 250 μL of the RPMI 1640 culture medium (Lonza, Basel, Switzerland) supplemented with 10% FBS (Sigma-Aldrich, Saint Louis, Missouri) and 1% penicillin-streptomycin (Lonza, Basel, Switzerland). Cells were cultured for 12 h at 37°C with 5% CO_2_ in the presence or absence of 9 × 10^3^ copies of EBV DNA. Triplicates of each treatment were performed. Cells were then collected and total RNA was extracted using Qiazol (Qiagen, Hilden, Germany) according to the manufacturer’s instructions. The isolated RNA was subsequently sequenced by Macrogen Inc. (Seoul, Korea) using the NovaSeq 6000 S4 Reagent Kit and an Illumina NovaSeq 6000 System (Illumina Inc., San Diego, CA, United States). Reads were trimmed with Trimmomatic and the trimmed reads were mapped to the *Mus musculus* mm10 reference genome using HISAT2; featureCounts was then used to assign sequence reads to genomic features and Limma-Voom was subsequently employed for differential expression (DE) analysis.

To visualize and analyse functional association network interactions involving the DEGs, GeneMANIA (Warde-Farley et al., 2010) using Cytoscape v3.8.2 was employed setting the max resultant genes to 20 and max resultant attributes to 10. Gene set enrichment (GSE) was conducted using EGSEA (Alhamdoosh et al., 2017) which combines data from 12 enrichment algorithms allowing analyses across multiple gene set collections. EGSEA v1.10.0 and EGSEAdata v1.10.0 were used with the Wilkinson *p*-value combining method employed for this analysis.

### 2.2 Isolation of immune cell-populations and flow cytometry analyses

Four to 6 week-old female BALB/c mice were used to isolate immune cell populations. For isolation of DCs, B cells and T cells, mouse spleens were each digested in 10 mL of a digestion mix consisting of 10 mg/mL collagenase IV (Roche) and 10 mg/mL DNAseI (Sigma-Aldrich, Saint Louis, Missouri) for 1 h at 37°C with gentle shaking. Splenocytes were then passed through Corning^®^ 70 μm cell strainers and washed with 1X PBS (Sigma-Aldrich, Saint Louis, Missouri). Cells obtained per spleen were then incubated with 5 mL of RBC lysis buffer (Qiagen, Hilden, Germany) for 10 min at room temperature. After two additional washes with 1X PBS, cells were incubated in T25 flasks in complete RPMI for 24 h at 37°C in 5% CO_2_. Cells were subsequently washed with 1X PBS and enumerated using a TC20™ Automated Cell Counter (Bio-Rad). To isolate elicited macrophages from peritoneal compartments, mice were each injected with 2.5 mL of 3% Brewer’s Thioglycolate medium (Scharlau, Barcelona, Spain) in the peritoneal cavity. After 4 days, mice were sacrificed and a small incision along the mouse midline was introduced with sterile scissors. The intact peritoneal wall was exposed and about 10 mL of cold sterile 1X PBS per mouse was injected in the cavity. The compartment was massaged for 5 min and the peritoneal fluid was then aspirated and centrifuged at 400 *g* for 10 min at 4°C. After two washes with 1X PBS, cells were resuspended in DMEM/F12-10 medium (Lonza, Basel, Switzerland) supplemented with 10% FBS (Sigma-Aldrich, Saint Louis, Missouri) and 1% penicillin-streptomycin (Lonza, Basel, Switzerland) and enumerated. A total of 10^7^ cells per well were cultured in 6-well plates in 2 mL of DMEM/F12-10 medium (Lonza, Basel, Switzerland) supplemented with 10% FBS and 1% penicillin-streptomycin (Lonza, Basel, Switzerland) then incubated overnight at 37°C in 5% CO_2_.

To isolate T cells, B cells, DCs, and macrophages, Fluorescence Activated Cell Sorting (FACS) was performed using a BD FACSAria™ II-SORP (BD Biosciences, San Jose, CA). For total leukocytes, an anti-mouse CD45 labelled with APC/Cy7 (BioLegend Inc. San Diego, CA) was employed. Then, for total T cells, an anti-mouse CD3 labelled with Alexa Fluor^®^ 647 (BioLegend Inc. San Diego, CA) was used. For B cells, PE-labelled anti-CD19 (BioLegend Inc. San Diego, CA) was employed and an anti-CD11c labelled with Brilliant Violet 421™ (BioLegend Inc. San Diego, CA) was used to collect pan DCs including plasmacytoid and myeloid DCs. The antibodies used for sorting of macrophages from the peritoneal exudate included anti-mouse CD11b and F4/80 labelled with PE and Brilliant Violet 421™ (BioLegend Inc. San Diego, CA) respectively.

### 2.3 Cell cultures and treatments

Isolated mouse B cells, DCs and macrophages were cultured each alone or with T cells in 96-well plates. Each APC population was co-cultured with T cells at a ratio of 1:10 which was found to be an optimal ratio in preliminary experiments for DCs. A similar ratio was then used for the other APC types to allow potency comparisons across cell types assessed. Thus, 15,000 of each APC type was cultured with 150,000 T cells per well in a total volume of 250 µL of complete RPMI. Mouse PBMCs (25 × 10^4^ per well) were also examined. Cells were either left untreated or cultured in the presence of 9 × 10^3^ copies of EBV DNA or 9 × 10^3^ EBV virions. The viral DNA was isolated from a P3HR-1 strain of EBV, which was also the strain used for testing the effects of the EBV viral particles. *S. epidermidis DNA* (1.7pg, equivalent to the weight of 9 × 10^3^ copies of EBV DNA) was used as a non-viral control DNA. Transcriptional analyses were conducted after 12 h of culture while supernatant levels of mediators were assayed 24 h after incubation.

For flow cytometry analysis of IL-17A positive cells, 25 × 10^4^ PBMCs were treated with 9 × 10^3^ copies of EBV DNA, 1.7 pg of *S. epidermidis* DNA, or mock treated (with culture medium). After 24 h of incubation cells were stained with anti-mouse CD3 labelled with Alexa Fluor^®^ 647 (BioLegend Inc. San Diego, CA) and anti-mouse IL-17A labelled with Brilliant Violet 605™ (BioLegend Inc. San Diego, CA) then analyzed by flow cytometry.

### 2.4 Enzyme-linked immunosorbent assay (ELISA)

Mouse IL-17A, IFNγ and TNF-α ELISA Kits (Abcam, Cambridge, United Kingdom) were used to determine culture supernatant levels of these mediators. Kits were used as per the manufacturer’s instructions.

### 2.5 Real-time PCR

To determine the relative expression of the IL-17A, IL-21, IL-23a, IFNγ, TNF-α, IL-12b, TLR9, TLR3, and CTLA4 genes, total RNA was isolated using Qiazol (Qiagen, Hilden, Germany) according to the manufacturer’s instructions. cDNA was synthesized employing the QuantiTect Reverse Transcription Kit (Qiagen, Hilden, Germany) and then real time RT-PCR was carried out using iTaq Universal SYBR Green Supermix (Bio-Rad) and employing the Bio-Rad CFX96 Real Time PCR Detection System (Bio-Rad). β-actin expression was used for expression normalization per sample. Previously published primers for IL-21 (Pesce et al., 2006), IL-23a (Teng et al., 2014), IL-12b (Sun et al., 2017), IL-17A, IFNγ, TNF-α, TLR9, TLR3, CTLA4, and β-actin (Andari et al., 2021) were used; primers were obtained from Macrogen Inc.

### 2.6 Statistical analysis

Statistical analyses for ELISA and real-time PCR were performed using GraphPad Prism. Means were compared using the two-tailed paired Student’s t-test. *p*-values less than 0.05 were considered statistically significant.

## 3 Results

### 3.1 Transcriptional profile of EBV DNA-treated mouse peripheral blood mononuclear cells

**FIGURE 1:**
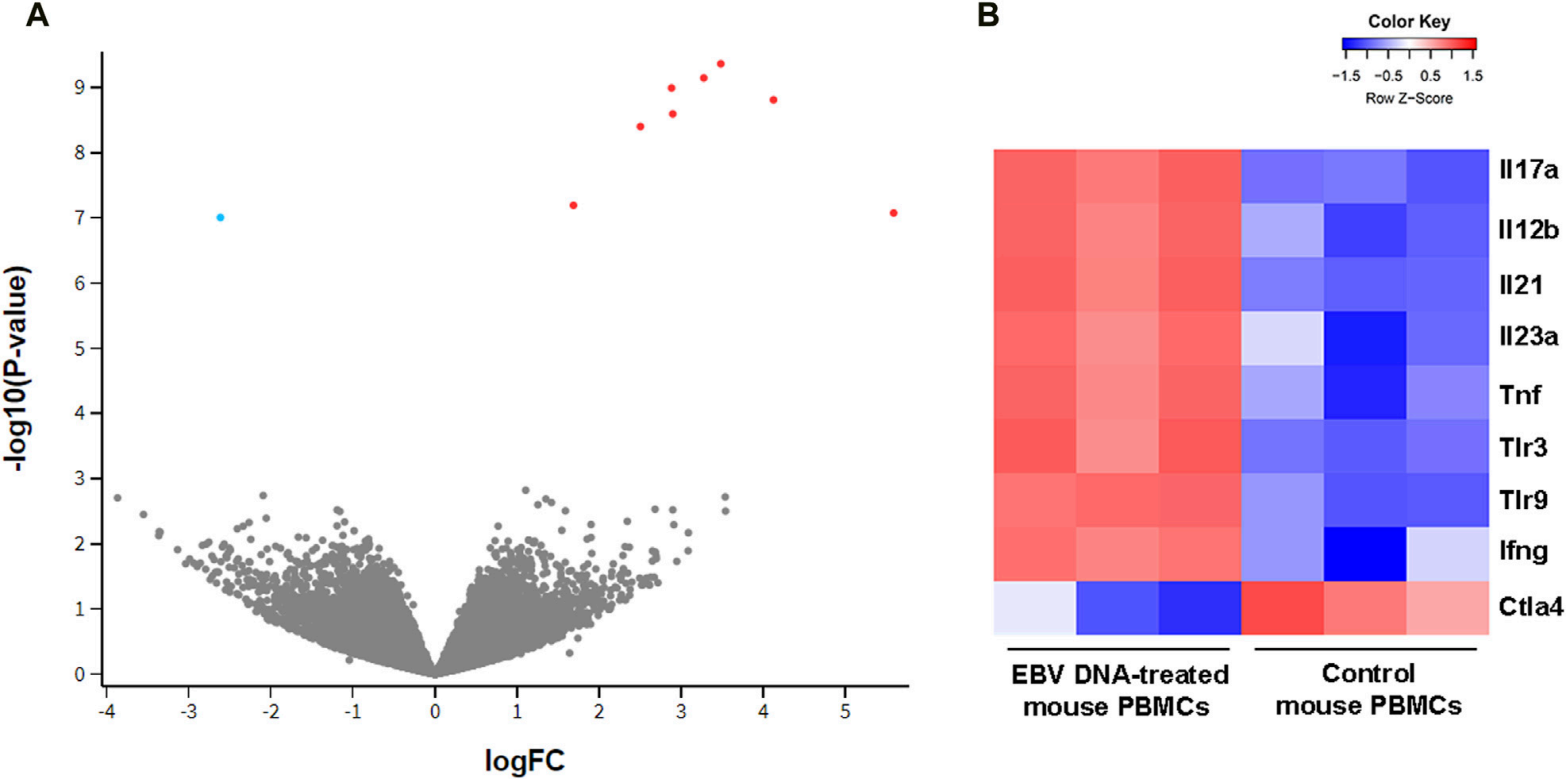
Transcriptional profile of mouse peripheral blood mononuclear cells (PBMCs) cultured in the presence of EBV DNA. Mouse PBMCs were cultured in the presence of viral DNA and their transcriptional profile was analyzed by RNA-seq compared to that of control PBMCs that were not treated with EBV DNA. **(A)** Volcano plot indicating significant differentially expressed genes (DEGs); red dots represent upregulated genes and blue dots represent downregulated ones. **(B)** Heat map representing the expression pattern of significant DEGs in samples of mouse PBMCs treated with EBV DNA; color shade indicates the Z-score with red representing increased expression and blue representing decreased expression.

To examine the effect of EBV DNA on the transcriptional profile of mouse peripheral blood mononuclear cells (PBMCs), cells were cultured in the presence or absence of the viral DNA then subjected to RNA extraction followed by RNA-seq. Analysis of differentially-expressed genes (DEGs) revealed a total of nine genes whose expression was significantly altered by the EBV DNA treatment. The expression of eight genes, including IL-17A, IFNγ, IL-21 and TNF-α, was significantly increased in the EBV DNA-treated group and one gene, CTLA4, showed a significant decrease in expression. Figure 1A is a volcano plot representing gene expression comparisons and Figure 1B is a heat map of the DEGs.

**FIGURE 2:**
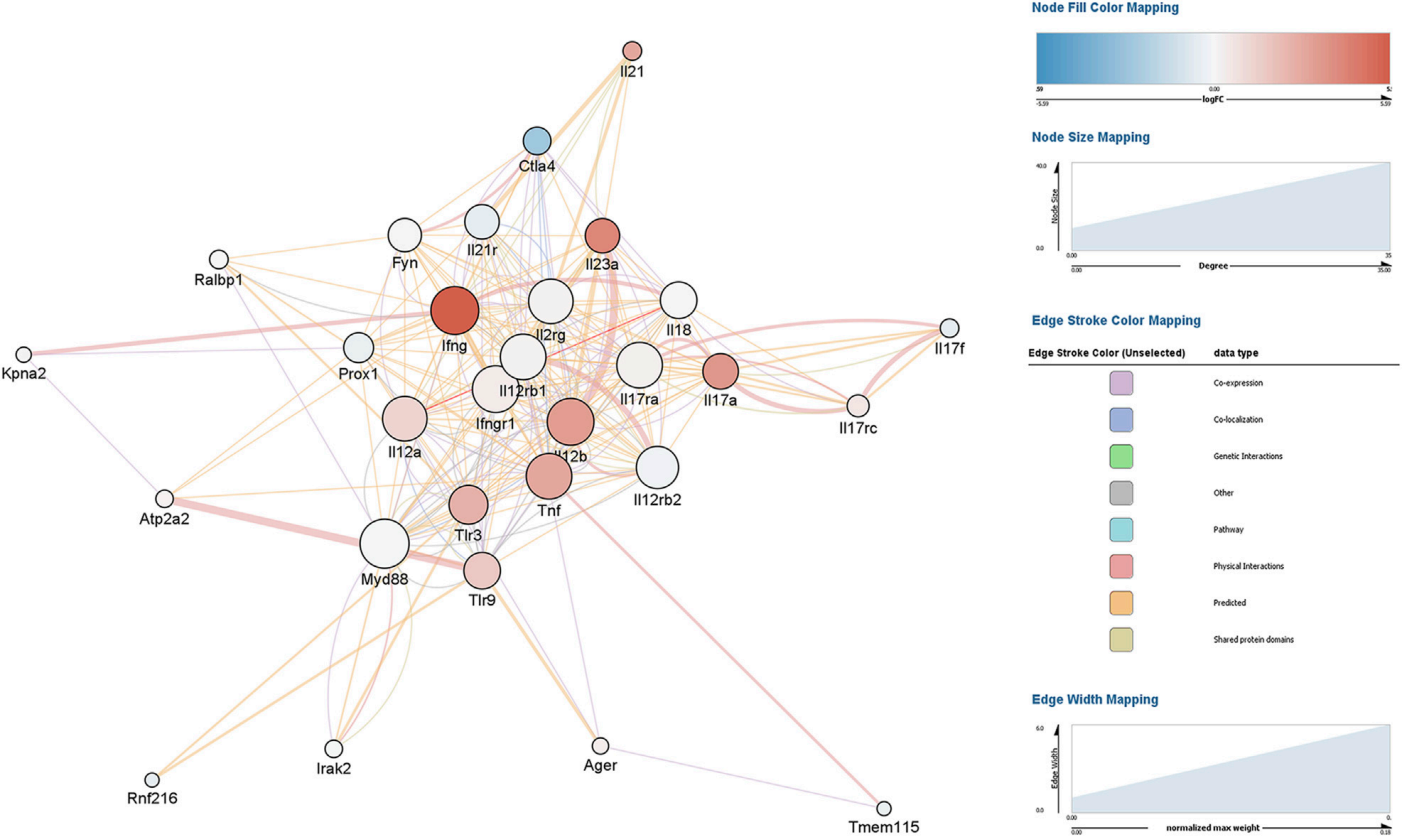
Gene interaction network analysis of differentially expressed genes (DEGs) in mouse peripheral blood mononuclear cells (PBMCs) cultured in the presence of EBV DNA. Mouse PBMCs were cultured in the presence or absence of viral DNA and their transcriptional profile was analyzed by RNA-seq. A gene interaction network for the DEGs was constructed and analyzed with GeneMANIA using Cytoscape.

Constructing an interaction gene network with GeneMANIA using Cytoscape to predict interactions between DEGs and other genes resulted in a network of 29 genes and 264 interaction edges (Figure 2). Node degrees were calculated to identify hub genes in the network; this analysis indicated that MyD88 followed closely by IFNγ were hub genes with the highest degree of connectivity in this module (Table 1).

**TABLE 1:** Node characteristics of the EBV DNA gene response network identified with GeneMANIA ranked by degree.

Gene name	Degree	Log score	Neighborhood connectivity	Score	Stress	Topological coefficient
Myd88	33	−4.088	15.529	0.017	184	0.555
Ifng	32	−0.610	14.350	0.543	226	0.513
Ifngr1	31	−2.984	17.063	0.051	46	0.609
Il12b	31	−0.350	16.500	0.705	76	0.589
Il12rb1	30	−3.029	15.882	0.048	100	0.567
Il17ra	30	−3.211	15.263	0.040	218	0.545
Tnf	30	−0.550	14.571	0.577	242	0.538
Il2rg	29	−3.854	16.056	0.021	96	0.573
Il12a	29	−4.250	15.368	0.014	174	0.549
Il12rb2	27	−3.790	16.882	0.023	54	0.603
Tlr3	24	−0.394	14.824	0.675	256	0.529
Tlr9	22	−0.541	14.077	0.582	124	0.521
Il18	22	−3.659	16.059	0.026	132	0.574
Il17a	21	−0.524	15.529	0.592	176	0.555
Il23a	20	−0.325	16.786	0.723	40	0.646
Il21r	20	−3.256	15.615	0.039	62	0.558
Fyn	19	−4.307	16.063	0.013	74	0.574
Prox1	16	−4.182	15.600	0.015	96	0.557
Ctla4	14	−0.384	17.727	0.681	10	0.682
Il17rc	9	−2.618	14.000	0.073	4	0.700
Il21	6	−0.314	16.800	0.731	6	0.730
Il17f	6	−3.602	13.333	0.027	0	0.702
Ralbp1	6	−4.209	18.200	0.015	2	0.728
Irak2	5	−3.978	17.000	0.019	0	0.895
Atp2a2	5	−2.468	14.400	0.085	14	0.576
Ager	4	−3.813	12.750	0.022	10	0.568
Kpna2	3	−3.543	13.333	0.029	0	0.606
Tmem115	2	−4.035	12.500	0.018	4	0.575
Rnf216	2	−3.618	15.000	0.027	0	0.714

**FIGURE 3:**
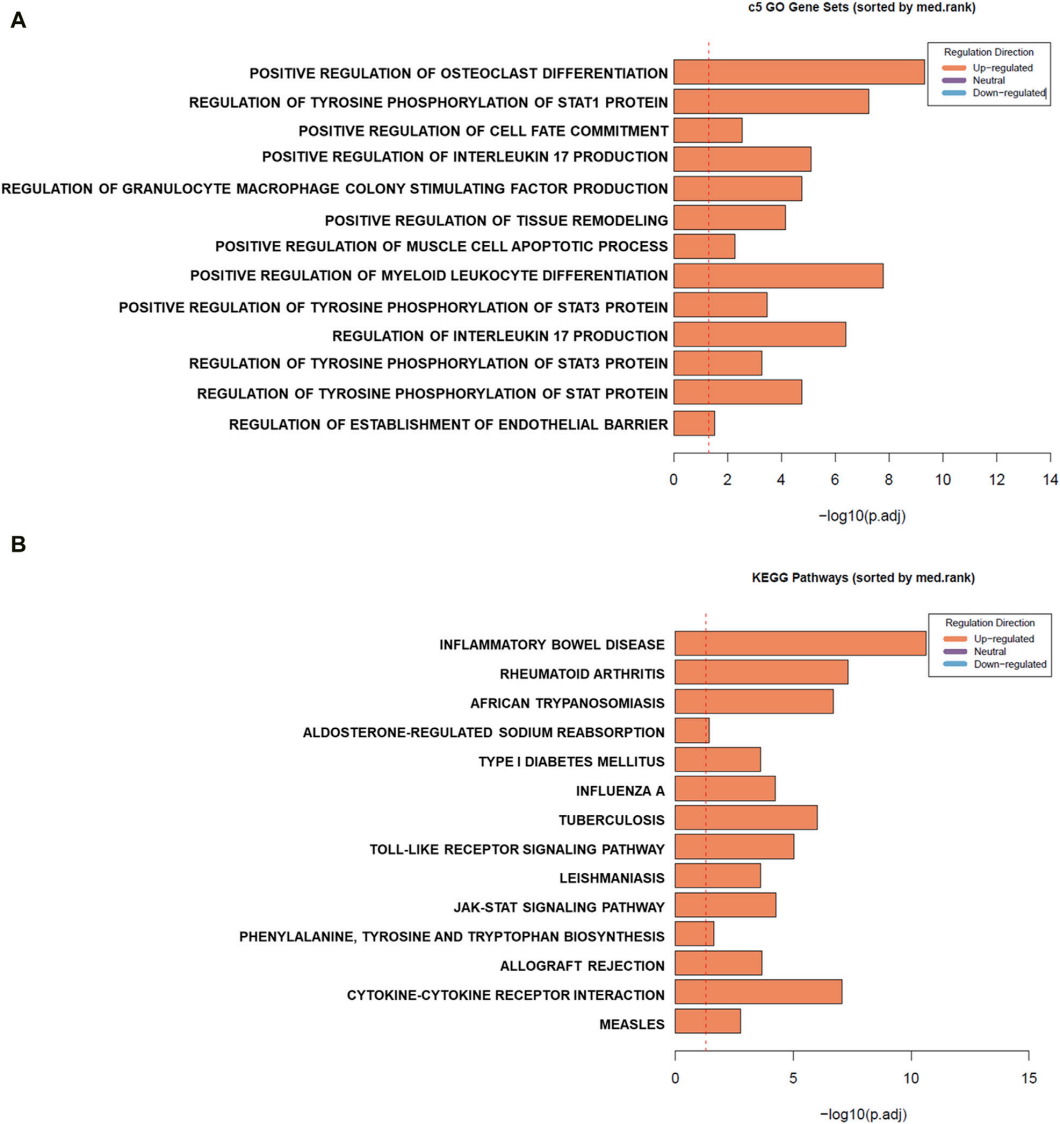
Gene set enrichment (GSE) analysis of differentially expressed genes (DEGs) in mouse peripheral blood mononuclear cells (PBMCs) cultured in the presence of EBV DNA. GSE was conducted using Ensemble Gene Set Enrichment Analysis (EGSEA). Summary plots based on gene set rank and size are indicated for sets in the Human MSigDB c5 gene ontology **(A)** and Kyoto Encyclopedia of Genes and Genomes (KEGG) **(B)** collections.

To assess the enrichment of gene sets detected to be differentially expressed in mouse PBMCs across human gene sets, Ensemble Gene Set Enrichment Analysis (EGSEA) was conducted (Figure 3) first using the Human MSigDB c5 gene ontology collection. We observed enrichment of gene sets involved mainly in inflammatory pathways such as those involved in positive regulation of IL-17 production, regulation of granulocyte macrophage colony stimulating factor production, positive regulation of myeloid leukocyte differentiation, and positive regulation of tissue remodelling. On the other hand, analysis using the Kyoto Encyclopedia of Genes and Genomes (KEGG) collections revealed enrichment of gene sets involved in proinflammatory diseases such as inflammatory bowel disease, rheumatoid arthritis, and type 1 diabetes mellitus, all of which are diseases associated with EBV infections.

**FIGURE 4:**
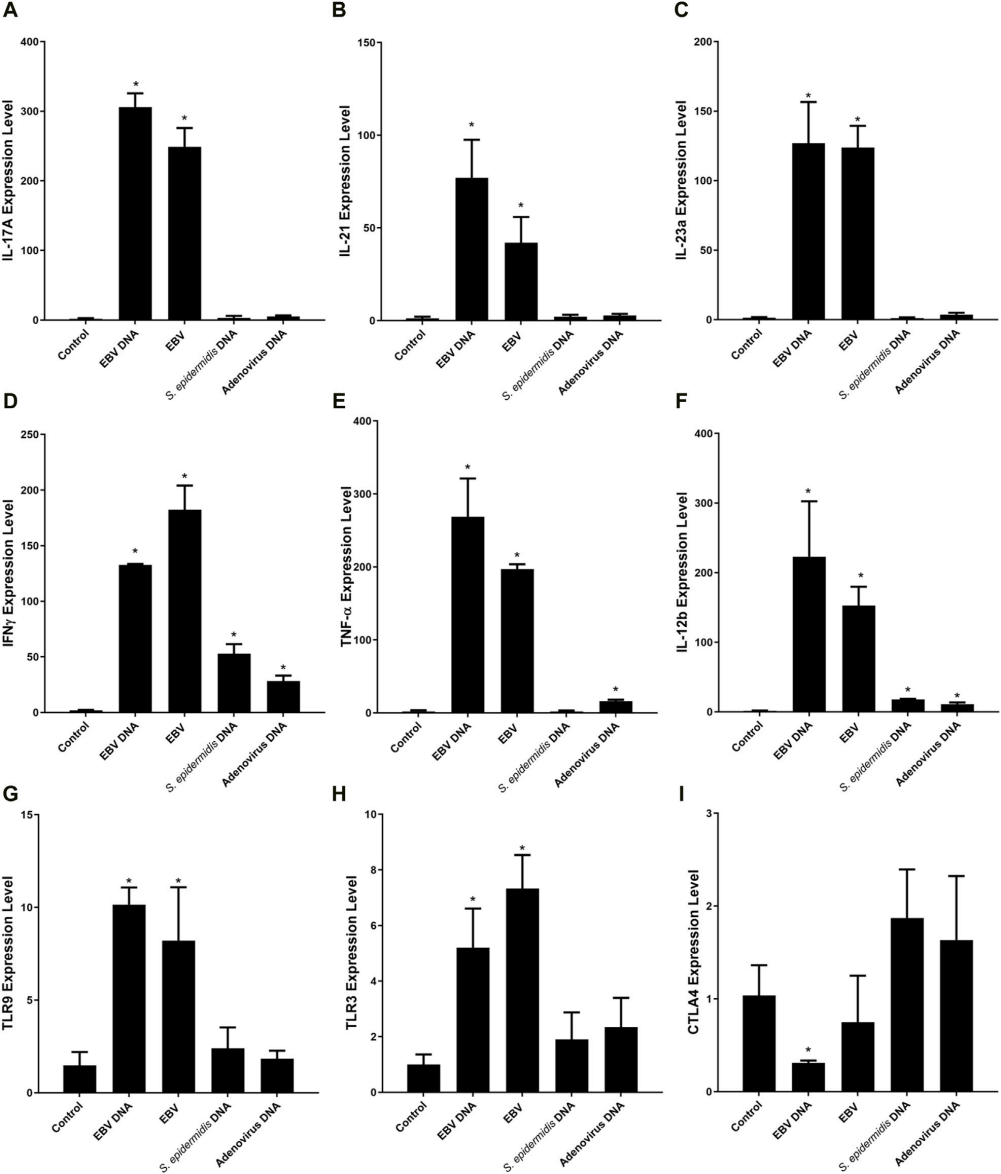
Relative expression of IL-17A **(A)**, IL-21 **(B)**, IL-23a **(C)**, IFNγ **(D)**, TNF-α **(E)**, IL-12b **(F)**, TLR9 **(G)**, TLR3 **(H)** and CTLA4 **(I)** in mouse peripheral blood mononuclear cells (PBMCs) cultured with EBV DNA. Mouse PBMCs were cultured in the presence or absence of viral DNA and relative expression levels were assessed with real-time PCR. The effects of bacterial control DNA from *S. epidermidis* and viral control DNA from human adenovirus type 1 C were assessed as well. * indicates *p* < 0.05 compared to non-treated PBMCs.

To validate the RNA-seq differential expression signature observed, the expression of all 9 DEGs was assessed in PBMCs treated with EBV DNA (Figure 4). Results paralleled the RNA-seq observations with enhanced expression of IL-17A, IL-21, IL-23a, IFNγ, TNF-α, IL-12b, TLR9 and TLR3 but a decreased expression of CTLA4. Culturing PBMCs with the virus itself also resulted in a similar expression pattern. Treatment with control bacterial DNA from *S. epidermidis* was able to enhance the expression of IFNγ and IL-12b, albeit not to the extent induced by EBV DNA; the bacterial DNA was not able to increase the expression of the other genes assessed. To examine the effects of another mammalian DNA virus, we assessed those of human adenovirus type 1 subgroup C; this viral DNA was also not able to replicate the expressional signature of EBV DNA. It was able to enhance the expression of IFNγ, TNF-α and IL-12b but to a much lesser extent compared to EBV DNA and it was not able to increase the expression of the other genes. This indicates that this IL-17A-centric signature is not induced by just any type of DNA and is likely more unique to EBV, and possibly to other herpesviruses as well.

### 3.2 Dendritic cell-T cell interactions drive the IL-17A-centric response to EBV DNA

Analysis of the proinflammatory network of genes triggered by EBV DNA indicated IL-17A to be one of the most highly upregulated genes with MyD88 seemingly a hub gene in this interaction network. With MyD88 being an adapter protein integral to TLR signalling, the role we observed for endosomal TLRs in the response to EBV DNA and the types of cytokines observed to be upregulated, T cell stimulation by innate immune responses to the viral DNA was a likely scenario. Hence, we sought to determine whether professional antigen presenting cells (APCs) mediate such a stimulation. Therefore, we examined the three types of professional APCs, B cells, dendritic cells (DCs) and macrophages; these cells were cultured alone or with T cells in the presence or absence of EBV DNA. This was followed by IL-17A level assessment in culture supernatants.

**FIGURE 5:**
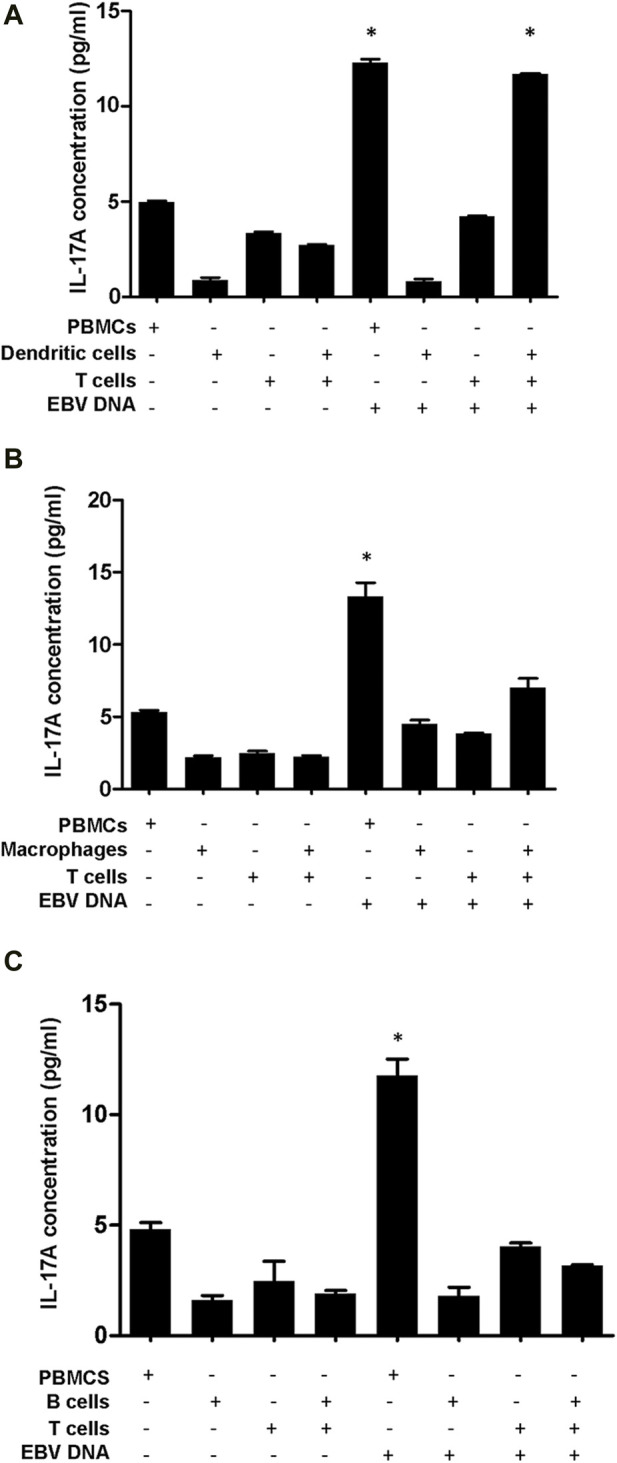
IL-17A levels in supernatants from co-cultures of mouse T cells and dendritic cells **(A)**, macrophages **(B)** or B cells **(C)** in the presence of EBV DNA. Peripheral blood mononuclear cells (PBMCs) cultured alone or in the presence of EBV DNA were included. * indicates *p* < 0.05 compared to non-treated PBMCs.

IL-17A levels were found to be significantly increased by 4.28 folds (*p* = 0.0001) in supernatants from mouse DCs and T cells co-cultured in the presence of EBV DNA compared to co-cultures of DCs and T cells without EBV DNA (Figure 5A). This co-culture of DCs and T cells with EBV DNA had a level similar to that of PBMCs cultured with EBV DNA; this PBMC control culture showed a 2.46-fold increase (*p* = 0.0006) in IL-17A levels compared to PBMCs cultured in the absence of the viral DNA. Mouse peritoneal elicited macrophages co-cultured with T cells showed a 3.12-fold increase in IL-17A levels upon treatment with EBV DNA compared to elicited macrophages and T cells co-cultured without EBV DNA (*p* = 0.0095) (Figure 5B). However, this increased level was 47.5% less than that observed in supernatants from PBMCs cultured in the presence of EBV DNA and hence not significant when compared to it (*p* = 0.0715). On the other hand, B cells co-cultured with T cells in the presence of EBV DNA resulted in some increase in IL-17A levels compared to a co-culture of these cell populations in the absence of EBV DNA; however, the level was 73% less than that of PBMCs treated with EBV DNA (Figure 5C). This indicates that DCs are the most potent in inducing an IL-17A response from T cells in the presence of EBV DNA.

**FIGURE 6:**
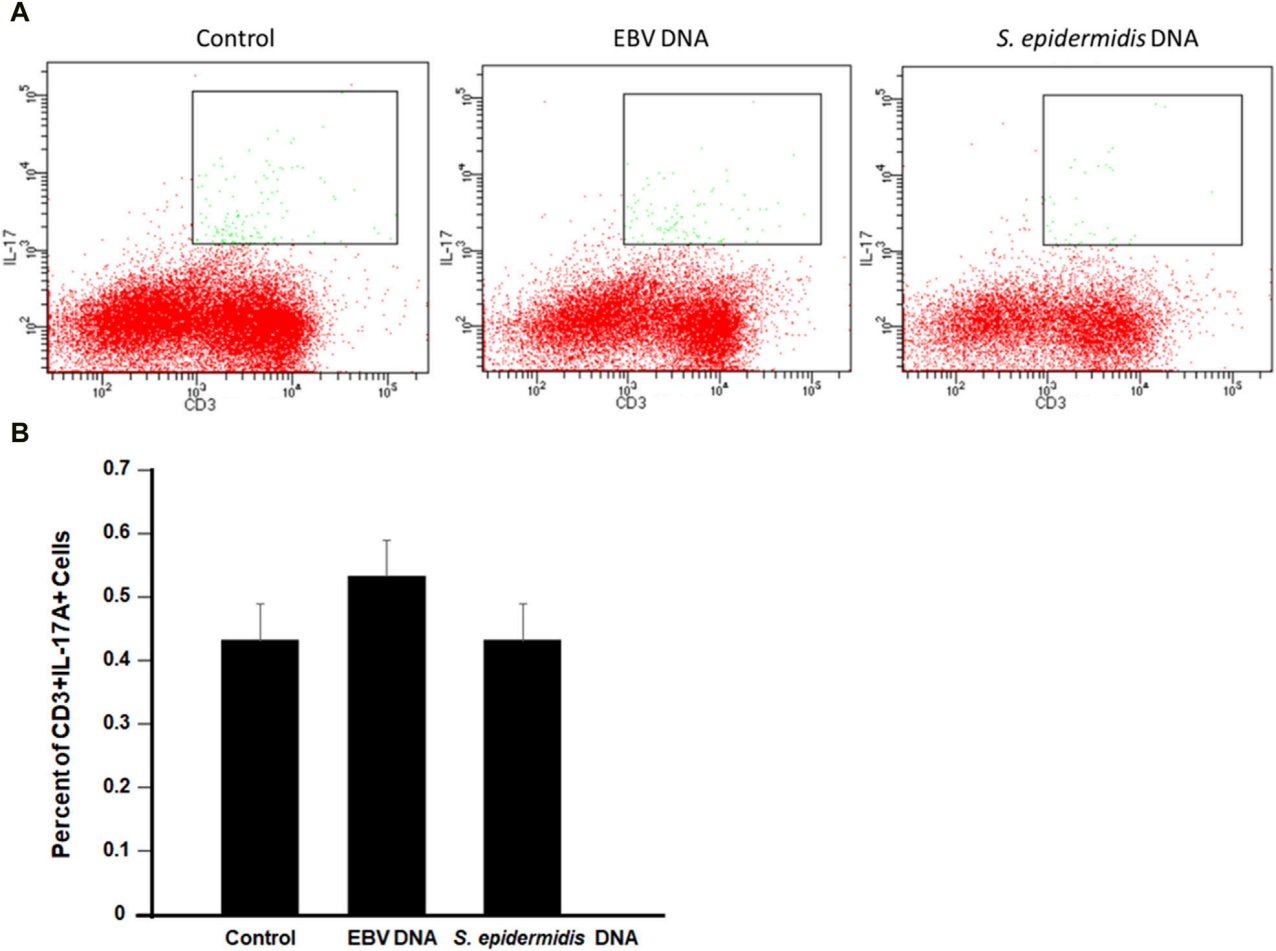
Flow cytometry analysis for CD3+IL-17A + cells in mouse peripheral blood mononuclear cells (PBMCs) cultured with EBV DNA. **(A)** Cells were cultured in the presence or absence of EBV DNA or control bacterial DNA from *S. epidermidis* then analyzed by flow cytometry. **(B)** Percent of CD3+IL-17A+ in PBMCs after treatment with the microbial DNA.

To examine whether the increased production of IL-17A upon EBV treatment of mouse immune cells was due to enhanced differentiation of T cells into Th17 cells, PBMCs were treated with EBV DNA, *S. epidermidis* DNA or mock treated with culture medium. Cells were subsequently analyzed by flow cytometry (Figure 6). We observed no marked differences between the different treatments which indicates that there is increased production of IL-17A from the EBV DNA-stimulated cells rather than enhanced differentiation into Th17 cells.

### 3.3 EBV virions trigger an IL-17A proinflammatory response from dendritic cell-T cell cultures

**FIGURE 7:**
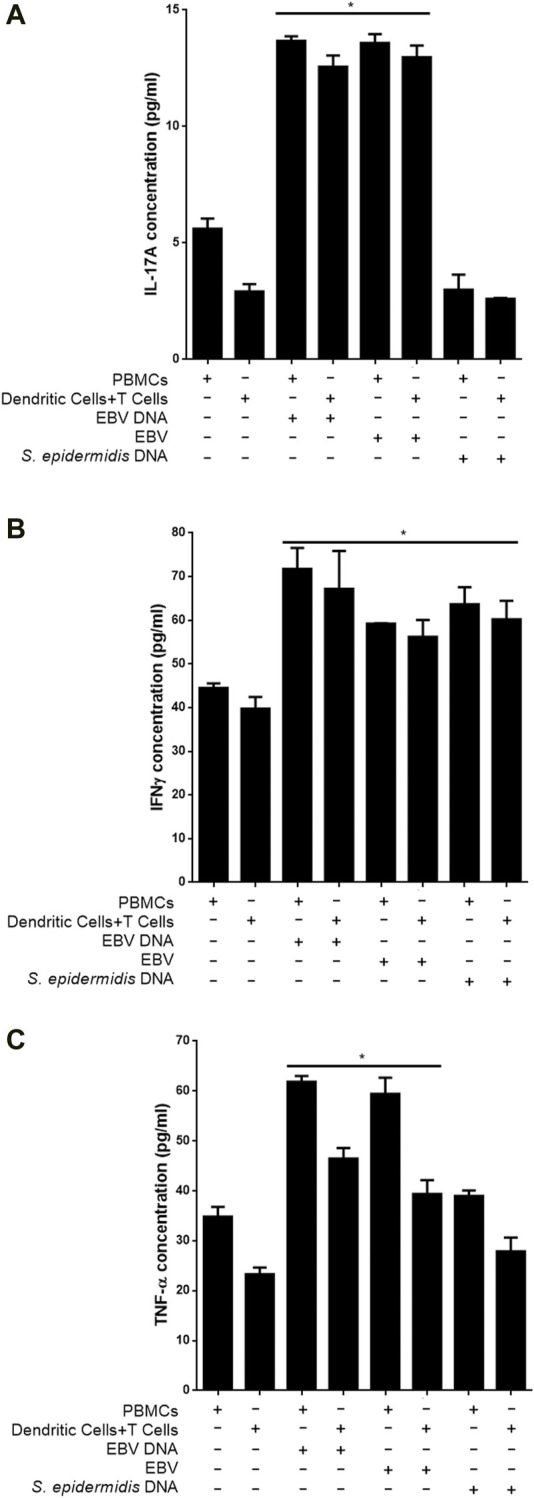
Proinflammatory mediator levels in supernatants from co-cultures of mouse T cells and dendritic cells in the presence of EBV. Cells were cultured with EBV, its DNA or with bacterial control DNA from *S. epidermidis*. Responses from mouse Peripheral blood mononuclear cells (PBMCs) were also assessed. Levels of IL-17A **(A)**, IFNγ **(B)** and TNF-α **(C)** were assessed in supernatants. * indicates *p* < 0.05 compared to respective non-treated cells.

To examine whether EBV particles are capable of triggering IL-17A production from DC and T cell co-cultures similar to the viral DNA, mouse cells were cultured with the viral particles. Subsequently, IL-17A levels were determined in culture supernatants (Figure 7A). We observed a 4.43-fold (*p* = 0.0017) increase in IL-17A levels from supernatants of DCs and T cells co-cultured in the presence of EBV compared to a co-culture of these cells in the absence of the virus. An increase of 2.42 folds (*p* = 0.0026) in IL-17A levels was also observed when PBMCs were cultured with the virus. Hence, the IL-17A response to the virus itself largely paralleled that to the viral DNA. On the other hand, control bacterial DNA from *S. epidermidis* was incapable of enhancing the production of IL-17A from PBMCs or DC-T cell co-cultures.

We also assessed whether other proinflammatory mediators observed to be triggered by EBV DNA were also triggered by the virus itself. Hence, IFNγ (Figure 7B) and TNF-α (Figure 7C) levels were examined in culture supernatants. A 41.43% increase in the level of IFNγ (*p* = 0.0383) was detected in supernatants from DC and T cell co-cultures in the presence of the virus compared to cells grown in the absence of the virus; PBMCs, on the other hand, showed a 32.92% (*p* = 0.0024) increase in IL-17A levels when cultured with EBV. EBV DNA was similarly capable of significantly increasing IFNγ from these cell types. Worth noting, is that unlike its inability to enhance production of IL-17A from PBMCs or DC-T cell co-cultures, *S. epidermidis* DNA was able to significantly increase IFNγ levels from these cells. Levels of TNF-α were also significantly elevated upon culturing PBMCs or DCs and T cells with the virus; similar observations were made in cultures of these cells with EBV DNA but not with *S. epidermidis* DNA.

## 4 Discussion

Infection with EBV has often been associated with autoimmune diseases including RA, SLE, MS, SS, mixed connective tissue disease, dermatomyositis/polymyositis and systemic scleroderma (Draborg et al., 2013). Various mechanisms have been proposed for how EBV infection potentiates autoimmune diseases. For example, multiple human nuclear antigens share epitope similarities with the EBV protein EBNA-1 and antibodies directed against EBNA-1 have been shown to cross react with double stranded DNA (Yadav et al., 2011) and with Ro, a component of several human ribonucleoprotein complexes (McClain et al., 2005). EBNA-1 was also shown to harbor epitopes that share similarities with myelin basic protein, the implicated autoantigen in multiple sclerosis, and with La antigen, which may trigger Sjögren’s syndrome, in addition to Sm (the small nuclear ribonucleoprotein) which may play a role in SLE (Poole et al., 2006; Kakalacheva et al., 2011; Carter, 2012). On the other hand, persistent inflammatory responses in infectious mononucleosis, caused by EBV, have been also been suggested to accidently result in the activation of auto-reactive lymphocytes hence resulting in autoimmunity (Lossius et al., 2012).

We have previously observed that EBV DNA, which is rich in immunostimulatory CpG motifs, enhances the production of IL-17A from mouse cells via endosomal TLR signaling (Shehab et al., 2019). IL-17A is a pro-inflammatory mediator that is prominently associated with autoimmunity. IL-17A is mostly produced by Th17 T-lymphocytes (Huber et al., 2013; Li et al., 2013). This cytokine was shown to increase T cell proliferation, antibody production from B cells and IL-1 and TNF-α secretion from macrophages (Bettelli et al., 2007; Iwakura et al., 2008). Furthermore, IL-17A can promote the release of myeloperoxidase from neutrophils (Zelante et al., 2007) and enhance expression of major histocompatibility complex II (MHC II) molecules on DCs (Banwell et al., 2007). In addition, IL-17A stimulates the release of IL-6 as well as IL-8 from keratinocytes and enhances the expression of these two cytokines by epithelial cells (Bettelli et al., 2007; Iwakura et al., 2008). Additionally, IL-17A is involved in neutrophil proliferation, maturation and chemotaxis processes. By stimulating the expression of inflammatory factors such as IL-6, IL-8, TNF-α and granulocyte-macrophage colony-stimulating factor (GM-CSF), chemokines like KC, MCP-1 and MIP-2 and matrix metalloproteases, IL-17A appears involved in enhancing tissue inflammation (Bettelli et al., 2007; Costa et al., 2010). IL-17A also triggers the expression of antimicrobial peptides such as β-defensin 2 (Liang et al., 2006). In *Candida albicans* infections IL-17A acts on epithelial cells inducing them to produce IL-8, a chemotactic factor for neutrophils which then counteract this fungal infection. In mice with a knocked out IL-17A receptor, a high fungal burden and a low survival rate were observed (Aggarwal and Gurney, 2002; Huang et al., 2004; Zelante et al., 2007). Contrary to its potentially beneficial effects in counteracting bacterial and fungal infections, IL-17A is believed to be involved in the pathogenesis of various autoimmune diseases. Mice with IL-17A or IL-17A receptor deficiency are less predisposed to experimental autoimmune encephalomyelitis (EAE) induction; these mice develop milder symptoms compared to non-deficient mice (Li et al., 2013; Qu et al., 2013). In autoimmune diseases, IL-17A seems to be often associated with another cytokine, a Th17 inducer, IL-23. Mice deficient in IL-23 or in IL-23 receptor are also not susceptible to EAE induction (Cua et al., 2003; McGeachy et al., 2009; Qu et al., 2013). IL-23 is essential for the survival and expansion of Th17 (Langrish et al., 2005; Bettelli et al., 2007; Burgler et al., 2009; McGeachy et al., 2009; Torchinsky et al., 2009; Qu et al., 2013). The differentiation of naïve T cells to Th17 requires RAR-related orphan receptor gamma (thymus) (RORγT), IFN-regulatory factor 4 (IRF4), aryl hydrocarbon receptor (AHR) and STAT3 in addition to TNF-α and IL-6 (Iwakura et al., 2008; Burgler et al., 2009; Costa et al., 2010; Qu et al., 2013). On the other hand, Th17 differentiation is negatively regulated by IFN-γ, IL-4, IL-2, type I IFN, IL-27 (Iwakura et al., 2008) and retinoic acid (Mucida et al., 2007). Although Th17 cells are known for their production of IL-17A, these cells can also express IL-22, IL-21, GM-CSF and potentially TNF-α and IL-1β (Qu et al., 2013). Hence, the network of proinflammatory mediators we detected to be involved in the response to EBV DNA involves ones that are associated with mediating IL-17A responses or that play a role in its regulation. On the other hand, we observed the downregulation of CTLA4 which is an immunoregulatory marker that suppresses T cell responses (Walker, 2013).

On the other hand, we observed upregulation of TLR3 and 9 with MyD88 detected to be a hub gene in our network analyses. TLRs are receptors that recognize pathogen-associated molecular patterns (PAMPs) and MyD88 is an adaptor protein that relays TLR signaling to downstream mediators. The TLR-MyD88 pathway has been implicated in autoimmune diseases (Devarapu and Anders, 2018; Zheng et al., 2019). Various pattern recognition receptors (PRRs) have been documented to respond to viral DNA; prominent among these are the Toll-like receptors (TLRs), but multiple other cellular receptors are potentially capable of detecting viral nucleic acids and these include DAI, RIG-I and AIM2 among many others [34]. We did not observe these to be transcriptionally upregulated in response to EBV DNA.

Expression of TLR9, the main TLR to respond to CpG rich DNA, in plasmacytoid DCs (pDCs) is the highest among other TLRs, Similarly, B cells and monocytic cells express TLR9; however, when compared to pDCs, they have lower TLR9 expression levels (Hornung et al., 2002). TLR9 was shown to be restricted to pDCs and not expressed in monocyte-derived DCs and CD11c+ DCs (Jarrossay et al., 2001; Kadowaki et al., 2001; Krug et al., 2001). In mouse secondary lymphoid tissues there are various DC types including CD11chi subsets like CD8α+, CD4^+^, the double-negative DCs, and the pDCs which are CD11clow (Vremec et al., 2000; Bjorck, 2001; Nakano et al., 2001). Endosomal TLRs are expressed by all these DC subsets with no significant differences between conventional CD11chi DCs and pDCs. In the study at hand, murine splenic DCs were sorted according to presence of the CD11c marker since we aimed at collecting all DC subsets. Splenic DCs co-cultured with T cell populations mirrored murine PBMC responses to EBV DNA. Hence, the high level of endosomal TLR expression by DCs likely underlies the potency of these cells to induce an IL-17A response from T-cells. While elicited peritoneal macrophages and splenic B cells were somewhat able to induce a response to EBV DNA, they were less potent in inducing T-cells to produce prominent levels of IL-17A.

On the other hand, our EGSEA analyses predict possible involvement of EBV DNA in IBD. We previously used a *Drosophila melanogaster* model to assess immune responses to EBV DNA (Sherri et al., 2018) and observed that this viral DNA enhances gut inflammatory responses in the fly model (Madi et al., 2021). We also observed enhanced IMD signaling in these flies; the IMD pathway is comparable to TNF receptor signaling in mammalian symptoms. We have similarly reported that EBV DNA exacerbates colitis in a mouse model of IBD (Andari et al., 2021). Hence, the roles played by this viral DNA in exacerbating or triggering inflammatory processes in IBD as well as other proinflammatory disease is worth further investigation.

Owing to the persistent nature of an EBV infection and its ability to cause recurrent infections, viral DNA that is shed would contribute to autoimmune processes on a more or less consistent basis. The EBV DNA cellular sensors along with downstream mediators and cells involved in the proinflammatory response unveiled by our study may serve as therapeutic targets in autoimmune and proinflammatory diseases. Very few studies have attempted to assess the burden of the more than 80 known autoimmune diseases. Conservative estimates in the United States indicate that 5%–8% of the population have an autoimmune disease (Autoimmune Diseases Coordinating Comittee, 2002). With more than 90% of the population being EBV seropositive, such therapeutic targets may be useful for the majority of people affected by an autoimmune disease.

## Data Availability

The RNAseq raw data discussed in this publication have been deposited in NCBI’s Gene Expression Omnibus (Edgar et al., 2002) and are accessible through GEO Series accession number GSE171757 (https://www.ncbi.nlm.nih.gov/geo/query/acc.cgi?acc=GSE171757). All other data is available from the authors upon reasonable request.

## References

[B1] Aggarwal S. Gurney A. L. (2002). IL-17: prototype member of an emerging cytokine family. J. Leukoc. Biol. 71 (1), 1–8. 10.1189/jlb.71.1.1 11781375

[B2] Alhamdoosh M. Ng M. Wilson N. J. Sheridan J. M. Huynh H. Wilson M. J. (2017). Combining multiple tools outperforms individual methods in gene set enrichment analyses. Bioinformatics 33 (3), 414–424. 10.1093/bioinformatics/btw623 27694195 PMC5408797

[B3] Andari S. Hussein H. Fadlallah S. Jurjus A. R. Shirinian M. Hashash J. G. (2021). Epstein-Barr virus DNA exacerbates colitis symptoms in a mouse model of inflammatory bowel disease. Viruses 13 (7), 1272. 10.3390/v13071272 34210024 PMC8310145

[B4] Ascherio A. Munger K. L. (2010). Epstein-barr virus infection and multiple sclerosis: a review. J. Neuroimmune Pharmacol. 5 (3), 271–277. 10.1007/s11481-010-9201-3 20369303

[B5] Autoimmune Diseases Coordinating Comittee (2002). “Autoimmune diseases research plan,” in Autoimmune diseases coordinating comittee. U.S. Department of health and human services (United States: National Institutes of Health NIH Publication).

[B6] Balandraud N. Meynard J. B. Auger I. Sovran H. Mugnier B. Reviron D. (2003). Epstein-Barr virus load in the peripheral blood of patients with rheumatoid arthritis: accurate quantification using real-time polymerase chain reaction. Arthritis Rheum. 48 (5), 1223–1228. 10.1002/art.10933 12746895

[B7] Banwell B. Krupp L. Kennedy J. Tellier R. Tenembaum S. Ness J. (2007). Clinical features and viral serologies in children with multiple sclerosis: a multinational observational study. Lancet Neurol. 6 (9), 773–781. 10.1016/S1474-4422(07)70196-5 17689148

[B8] Bettelli E. Oukka M. Kuchroo V. K. (2007). T(H)-17 cells in the circle of immunity and autoimmunity. Nat. Immunol. 8 (4), 345–350. 10.1038/ni0407-345 17375096

[B9] Bjorck P. (2001). Isolation and characterization of plasmacytoid dendritic cells from Flt3 ligand and granulocyte-macrophage colony-stimulating factor-treated mice. Blood 98 (13), 3520–3526. 10.1182/blood.v98.13.3520 11739152

[B10] Blaschke S. Schwarz G. Moneke D. Binder L. Muller G. Reuss-Borst M. (2000). Epstein-Barr virus infection in peripheral blood mononuclear cells, synovial fluid cells, and synovial membranes of patients with rheumatoid arthritis. J. Rheumatol. 27 (4), 866–873. 10782808

[B11] Burgler S. Ouaked N. Bassin C. Basinski T. M. Mantel P. Y. Siegmund K. (2009). Differentiation and functional analysis of human T(H)17 cells. J. Allergy Clin. Immunol. 123 (3), 588–595. 10.1016/j.jaci.2008.12.017 19178935

[B12] Carter C. J. (2012). Epstein-Barr and other viral mimicry of autoantigens, myelin and vitamin D-related proteins and of EIF2B, the cause of vanishing white matter disease: massive mimicry of multiple sclerosis relevant proteins by the Synechococcus phage. Immunopharmacol. Immunotoxicol. 34 (1), 21–35. 10.3109/08923973.2011.572262 21486137

[B13] Chen C. J. Lin K. H. Lin S. C. Tsai W. C. Yen J. H. Chang S. J. (2005). High prevalence of immunoglobulin A antibody against Epstein-Barr virus capsid antigen in adult patients with lupus with disease flare: case control studies. J. Rheumatol. 32 (1), 44–47. 15630723

[B14] Costa V. S. Mattana T. C. da Silva M. E. (2010). Unregulated IL-23/IL-17 immune response in autoimmune diseases. Diabetes Res. Clin. Pract. 88 (3), 222–226. 10.1016/j.diabres.2010.03.014 20392505

[B15] Cua D. J. Sherlock J. Chen Y. Murphy C. A. Joyce B. Seymour B. (2003). Interleukin-23 rather than interleukin-12 is the critical cytokine for autoimmune inflammation of the brain. Nature 421 (6924), 744–748. 10.1038/nature01355 12610626

[B16] Devarapu S. K. Anders H. J. (2018). Toll-like receptors in lupus nephritis. J. Biomed. Sci. 25 (1), 35. 10.1186/s12929-018-0436-2 29650017 PMC5898010

[B17] Draborg A. H. Duus K. Houen G. (2012). Epstein-Barr virus and systemic lupus erythematosus. Clin. Dev. Immunol. 2012, 370516. 10.1155/2012/370516 22811739 PMC3395176

[B18] Draborg A. H. Duus K. Houen G. (2013). Epstein-Barr virus in systemic autoimmune diseases. Clin. Dev. Immunol. 2013, 535738. 10.1155/2013/535738 24062777 PMC3766599

[B19] Edgar R. Domrachev M. Lash A. E. (2002). Gene Expression Omnibus: NCBI gene expression and hybridization array data repository. Nucleic Acids Res. 30 (1), 207–210. 10.1093/nar/30.1.207 11752295 PMC99122

[B20] Fiola S. Gosselin D. Takada K. Gosselin J. (2010). TLR9 contributes to the recognition of EBV by primary monocytes and plasmacytoid dendritic cells. J. Immunol. 185 (6), 3620–3631. 10.4049/jimmunol.0903736 20713890

[B21] Fox R. I. Chilton T. Scott S. Benton L. Howell F. V. Vaughan J. H. (1987). Potential role of Epstein-Barr virus in Sjogren's syndrome. Rheum. Dis. Clin. North Am. 13 (2), 275–292. 10.1016/s0889-857x(21)00847-4 2827246

[B22] Gross A. J. Hochberg D. Rand W. M. Thorley-Lawson D. A. (2005). EBV and systemic lupus erythematosus: a new perspective. J. Immunol. 174 (11), 6599–6607. 10.4049/jimmunol.174.11.6599 15905498

[B23] Hornung V. Rothenfusser S. Britsch S. Krug A. Jahrsdorfer B. Giese T. (2002). Quantitative expression of toll-like receptor 1-10 mRNA in cellular subsets of human peripheral blood mononuclear cells and sensitivity to CpG oligodeoxynucleotides. J. Immunol. 168 (9), 4531–4537. 10.4049/jimmunol.168.9.4531 11970999

[B24] Huang W. Na L. Fidel P. L. Schwarzenberger P. (2004). Requirement of interleukin-17A for systemic anti-Candida albicans host defense in mice. J. Infect. Dis. 190 (3), 624–631. 10.1086/422329 15243941

[B25] Huber M. Heink S. Pagenstecher A. Reinhard K. Ritter J. Visekruna A. (2013). IL-17A secretion by CD8+ T cells supports Th17-mediated autoimmune encephalomyelitis. J. Clin. Invest. 123 (1), 247–260. 10.1172/JCI63681 23221338 PMC3533283

[B26] Iwakiri D. (2014). Epstein-Barr virus-encoded RNAs: key molecules in viral pathogenesis. Cancers (Basel) 6 (3), 1615–1630. 10.3390/cancers6031615 25101570 PMC4190559

[B27] Iwakura Y. Nakae S. Saijo S. Ishigame H. (2008). The roles of IL-17A in inflammatory immune responses and host defense against pathogens. Immunol. Rev. 226, 57–79. 10.1111/j.1600-065X.2008.00699.x 19161416

[B28] James J. A. Kaufman K. M. Farris A. D. Taylor-Albert E. Lehman T. J. Harley J. B. (1997). An increased prevalence of Epstein-Barr virus infection in young patients suggests a possible etiology for systemic lupus erythematosus. J. Clin. Invest. 100 (12), 3019–3026. 10.1172/JCI119856 9399948 PMC508514

[B29] Jarrossay D. Napolitani G. Colonna M. Sallusto F. Lanzavecchia A. (2001). Specialization and complementarity in microbial molecule recognition by human myeloid and plasmacytoid dendritic cells. Eur. J. Immunol. 31 (11), 3388–3393. 10.1002/1521-4141(200111)31:11<3388::aid-immu3388>3.0.co;2-q 11745357

[B30] Kadowaki N. Ho S. Antonenko S. Malefyt R. W. Kastelein R. A. Bazan F. (2001). Subsets of human dendritic cell precursors express different toll-like receptors and respond to different microbial antigens. J. Exp. Med. 194 (6), 863–869. 10.1084/jem.194.6.863 11561001 PMC2195968

[B31] Kakalacheva K. Munz C. Lunemann J. D. (2011). Viral triggers of multiple sclerosis. Biochim. Biophys. Acta 1812 (2), 132–140. 10.1016/j.bbadis.2010.06.012 20600868 PMC7126972

[B32] Kang I. Quan T. Nolasco H. Park S. H. Hong M. S. Crouch J. (2004). Defective control of latent Epstein-Barr virus infection in systemic lupus erythematosus. J. Immunol. 172 (2), 1287–1294. 10.4049/jimmunol.172.2.1287 14707107

[B33] Kieff E. Dambaugh T. Heller M. King W. Cheung A. van Santen V. (1982). The biology and chemistry of Epstein-Barr virus. J. Infect. Dis. 146 (4), 506–517. 10.1093/infdis/146.4.506 6288806

[B34] Krug A. Towarowski A. Britsch S. Rothenfusser S. Hornung V. Bals R. (2001). Toll-like receptor expression reveals CpG DNA as a unique microbial stimulus for plasmacytoid dendritic cells which synergizes with CD40 ligand to induce high amounts of IL-12. Eur. J. Immunol. 31 (10), 3026–3037. 10.1002/1521-4141(2001010)31:10<3026::aid-immu3026>3.0.co;2-h 11592079

[B35] Langrish C. L. Chen Y. Blumenschein W. M. Mattson J. Basham B. Sedgwick J. D. (2005). IL-23 drives a pathogenic T cell population that induces autoimmune inflammation. J. Exp. Med. 201 (2), 233–240. 10.1084/jem.20041257 15657292 PMC2212798

[B36] Larsen M. Sauce D. Deback C. Arnaud L. Mathian A. Miyara M. (2011). Exhausted cytotoxic control of Epstein-Barr virus in human lupus. PLoS Pathog. 7 (10), e1002328. 10.1371/journal.ppat.1002328 22028659 PMC3197610

[B37] Li Z. Li K. Zhu L. Kan Q. Yan Y. Kumar P. (2013). Inhibitory effect of IL-17 on neural stem cell proliferation and neural cell differentiation. BMC Immunol. 14, 20. 10.1186/1471-2172-14-20 23617463 PMC3642028

[B38] Liang S. C. Tan X. Y. Luxenberg D. P. Karim R. Dunussi-Joannopoulos K. Collins M. (2006). Interleukin (IL)-22 and IL-17 are coexpressed by Th17 cells and cooperatively enhance expression of antimicrobial peptides. J. Exp. Med. 203 (10), 2271–2279. 10.1084/jem.20061308 16982811 PMC2118116

[B39] Lindsey J. W. Hatfield L. M. Crawford M. P. Patel S. (2009). Quantitative PCR for Epstein-Barr virus DNA and RNA in multiple sclerosis. Mult. Scler. 15 (2), 153–158. 10.1177/1352458508097920 18845656

[B40] Lossius A. Johansen J. N. Torkildsen O. Vartdal F. Holmoy T. (2012). Epstein-Barr virus in systemic lupus erythematosus, rheumatoid arthritis and multiple sclerosis-association and causation. Viruses 4 (12), 3701–3730. 10.3390/v4123701 23342374 PMC3528287

[B41] Lotz M. Roudier J. (1989). Epstein-Barr virus and rheumatoid arthritis: cellular and molecular aspects. Rheumatol. Int. 9 (3-5), 147–152. 10.1007/BF00271872 2481874

[B42] Lucas R. M. Hughes A. M. Lay M. L. Ponsonby A. L. Dwyer D. E. Taylor B. V. (2011). Epstein-Barr virus and multiple sclerosis. J. Neurol. Neurosurg. Psychiatry 82 (10), 1142–1148. 10.1136/jnnp-2011-300174 21836034

[B43] Lunemann J. D. Edwards N. Muraro P. A. Hayashi S. Cohen J. I. Munz C. (2006). Increased frequency and broadened specificity of latent EBV nuclear antigen-1-specific T cells in multiple sclerosis. Brain 129 (6), 1493–1506. 10.1093/brain/awl067 16569670

[B44] Lunemann J. D. Huppke P. Roberts S. Bruck W. Gartner J. Munz C. (2008). Broadened and elevated humoral immune response to EBNA1 in pediatric multiple sclerosis. Neurology 71 (13), 1033–1035. 10.1212/01.wnl.0000326576.91097.87 18809840 PMC2676958

[B45] Lunemann J. D. Kamradt T. Martin R. Munz C. (2007). Epstein-barr virus: environmental trigger of multiple sclerosis? J. Virol. 81 (13), 6777–6784. 10.1128/JVI.00153-07 17459939 PMC1933281

[B46] Madi J. R. Outa A. A. Ghannam M. Hussein H. M. Shehab M. Hasan Z. (2021). *Drosophila melanogaster* as a model System to assess the effect of epstein-barr virus DNA on inflammatory gut diseases. Front. Immunol. 12, 586930. 10.3389/fimmu.2021.586930 33828545 PMC8019809

[B47] McClain M. T. Heinlen L. D. Dennis G. J. Roebuck J. Harley J. B. James J. A. (2005). Early events in lupus humoral autoimmunity suggest initiation through molecular mimicry. Nat. Med. 11 (1), 85–89. 10.1038/nm1167 15619631

[B48] McGeachy M. J. Chen Y. Tato C. M. Laurence A. Joyce-Shaikh B. Blumenschein W. M. (2009). The interleukin 23 receptor is essential for the terminal differentiation of interleukin 17-producing effector T helper cells *in vivo* . Nat. Immunol. 10 (3), 314–324. 10.1038/ni.1698 19182808 PMC2945605

[B49] Mucida D. Park Y. Kim G. Turovskaya O. Scott I. Kronenberg M. (2007). Reciprocal TH17 and regulatory T cell differentiation mediated by retinoic acid. Science 317 (5835), 256–260. 10.1126/science.1145697 17569825

[B50] Nakano H. Yanagita M. Gunn M. D. (2001). CD11c(+)B220(+)Gr-1(+) cells in mouse lymph nodes and spleen display characteristics of plasmacytoid dendritic cells. J. Exp. Med. 194 (8), 1171–1178. 10.1084/jem.194.8.1171 11602645 PMC2193516

[B51] Pesce J. Kaviratne M. Ramalingam T. R. Thompson R. W. Urban J. F. Jr. Cheever A. W. (2006). The IL-21 receptor augments Th2 effector function and alternative macrophage activation. J. Clin. Invest. 116 (7), 2044–2055. 10.1172/JCI27727 16778988 PMC1479424

[B52] Petersen J. Rhodes G. Roudier J. Vaughan J. H. (1990). Altered immune response to glycine-rich sequences of Epstein-Barr nuclear antigen-1 in patients with rheumatoid arthritis and systemic lupus erythematosus. Arthritis Rheum. 33 (7), 993–1000. 10.1002/art.1780330711 2164400

[B53] Poole B. D. Scofield R. H. Harley J. B. James J. A. (2006). Epstein-Barr virus and molecular mimicry in systemic lupus erythematosus. Autoimmunity 39 (1), 63–70. 10.1080/08916930500484849 16455583

[B54] Qu N. Xu M. Mizoguchi I. Furusawa J. Kaneko K. Watanabe K. (2013). Pivotal roles of T-helper 17-related cytokines, IL-17, IL-22, and IL-23, in inflammatory diseases. Clin. Dev. Immunol. 2013, 968549. 10.1155/2013/968549 23956763 PMC3728507

[B55] Rahal E. A. Hajjar H. Rajeh M. Yamout B. Abdelnoor A. M. (2015). Epstein-Barr virus and human herpes virus 6 type A DNA enhance IL-17 production in mice. Viral Immunol. 28 (5), 297–302. 10.1089/vim.2014.0129 25870901

[B56] Salloum N. Hussein H. M. Jammaz R. Jiche S. Uthman I. W. Abdelnoor A. M. (2018). Epstein-Barr virus DNA modulates regulatory T-cell programming in addition to enhancing interleukin-17A production via Toll-like receptor 9. PLoS One 13 (7), e0200546. 10.1371/journal.pone.0200546 29995930 PMC6040775

[B57] Shehab M. Sherri N. Hussein H. Salloum N. Rahal E. A. (2019). Endosomal toll-like receptors mediate enhancement of interleukin-17a production triggered by epstein-barr virus DNA in mice. J. Virol. 93 (20), e00987-19. 10.1128/JVI.00987-19 31375581 PMC6798095

[B58] Sherri N. Salloum N. Mouawad C. Haidar-Ahmad N. Shirinian M. Rahal E. A. (2018). Epstein-Barr virus DNA enhances diptericin expression and increases hemocyte numbers in *Drosophila melanogaster* via the immune deficiency pathway. Front. Microbiol. 9, 1268. 10.3389/fmicb.2018.01268 29942298 PMC6004391

[B59] Shirodaria P. V. Haire M. Fleming E. Merrett J. D. Hawkins S. A. Roberts S. D. (1987). Viral antibody titers. Comparison in patients with multiple sclerosis and rheumatoid arthritis. Arch. Neurol. 44 (12), 1237–1241. 10.1001/archneur.1987.00520240019006 2823754

[B60] Sun J. Huang P. Liang J. Li J. Shen M. She X. (2017). Cooperation of Rel family members in regulating Aβ1-40-mediated pro-inflammatory cytokine secretion by retinal pigment epithelial cells. Cell Death Dis. 8 (10), e3115. 10.1038/cddis.2017.502 29022897 PMC5682668

[B61] Teng X. Hu Z. Wei X. Wang Z. Guan T. Liu N. (2014). IL-37 ameliorates the inflammatory process in psoriasis by suppressing proinflammatory cytokine production. J. Immunol. 192 (4), 1815–1823. 10.4049/jimmunol.1300047 24453242

[B62] Torchinsky M. B. Garaude J. Martin A. P. Blander J. M. (2009). Innate immune recognition of infected apoptotic cells directs T(H)17 cell differentiation. Nature 458 (7234), 78–82. 10.1038/nature07781 19262671

[B63] Tosato G. Steinberg A. D. Yarchoan R. Heilman C. A. Pike S. E. De Seau V. (1984). Abnormally elevated frequency of Epstein-Barr virus-infected B cells in the blood of patients with rheumatoid arthritis. J. Clin. Invest. 73 (6), 1789–1795. 10.1172/JCI111388 6327772 PMC437092

[B64] Vremec D. Pooley J. Hochrein H. Wu L. Shortman K. (2000). CD4 and CD8 expression by dendritic cell subtypes in mouse thymus and spleen. J. Immunol. 164 (6), 2978–2986. 10.4049/jimmunol.164.6.2978 10706685

[B65] Wagner H. J. Munger K. L. Ascherio A. (2004). Plasma viral load of Epstein-Barr virus and risk of multiple sclerosis. Eur. J. Neurol. 11 (12), 833–834. 10.1111/j.1468-1331.2004.00871.x 15667414

[B66] Walker L. S. (2013). Treg and CTLA-4: two intertwining pathways to immune tolerance. J. Autoimmun. 45, 49–57. 10.1016/j.jaut.2013.06.006 23849743 PMC3989116

[B67] Wandinger K. Jabs W. Siekhaus A. Bubel S. Trillenberg P. Wagner H. (2000). Association between clinical disease activity and Epstein-Barr virus reactivation in MS. Neurology 55 (2), 178–184. 10.1212/wnl.55.2.178 10908887

[B68] Warde-Farley D. Donaldson S. L. Comes O. Zuberi K. Badrawi R. Chao P. (2010). The GeneMANIA prediction server: biological network integration for gene prioritization and predicting gene function. Nucleic Acids Res. 38, W214–W220. 10.1093/nar/gkq537 20576703 PMC2896186

[B69] Yadav P. Tran H. Ebegbe R. Gottlieb P. Wei H. Lewis R. H. (2011). Antibodies elicited in response to EBNA-1 may cross-react with dsDNA. PLoS One 6 (1), e14488. 10.1371/journal.pone.0014488 21245919 PMC3014975

[B70] Zelante T. De Luca A. Bonifazi P. Montagnoli C. Bozza S. Moretti S. (2007). IL-23 and the Th17 pathway promote inflammation and impair antifungal immune resistance. Eur. J. Immunol. 37 (10), 2695–2706. 10.1002/eji.200737409 17899546

[B71] Zheng C. Chen J. Chu F. Zhu J. Jin T. (2019). Inflammatory role of TLR-MyD88 signaling in multiple sclerosis. Front. Mol. Neurosci. 12, 314. 10.3389/fnmol.2019.00314 31998072 PMC6965019

